# The Foreign Journals: Surgery

**Published:** 1840-10

**Authors:** 


*Bulletin de l'Académie Royale de Médecine. Fevrier* 29, 1840.

### SURGERY.

### *On a New Species of Strangulation in the Tunica Vaginalis Testis, followed by Internal Strangulation.* By M. Laugier.

M. Laugier, surgeon to the hospital Beaujon, presented to the academy a pathological specimen, which is an extraordinary example of the above strangulation, caused by a circular fold of peritoneum, the edge of which surrounds the superior orifice of the inguinal canal and the free border of which floats in the abdomen, forming a sort of prolongation of the inguinal canal. It was formed by a double fold of peritoneum detached from the anterior wall of the abdomen. It was easily passed into the tunica vaginalis (like the finger of a glove cut off) from ten to twelve lines. It was returned with equal facility. A portion of small intestine, about a foot in length, had pushed this production before it in passing into the sac of the tunica vaginalis, and became strangulated by the floating ring. The strangulation, therefore, was not at the neck of the sac, as usually occurs in congenital hernia; neither was it an encysted congenital hernia, for there was no secondary sac, but a ring at the free part of the floating fold. The reduction of the strangulated hernia produced the separation of the circular band which surrounded the intestine, and the external strangulation became internal, without there being any possibility of suspecting before death a similar course of strangulation in the tunica vaginalis and internal strangulation.

The dissection revealed the existence of a similar fold on the opposite side, and the patient had a congenital reducible hernia in the left groin. But as there was neither strangulation nor rupture, the free border of the fold was rounded and not broken as on the right side. No similar disposition of parts has been hitherto recorded. If it had been possible to suspect it during life it is not likely that an attempt at reduction would have been made, for the reduction itself was the cause of the strangulation in producing rupture of the fold. Gastrotomy was the only operation that could have been practised, but the rapid death of the patient prevented this.

*Bulletin de l'Académie Royale de Médecine. Juin* 30, 1840.

### *Peritonitis from Injections into the Uterus, the Injections passing through the Fallopian Tubes into the Cavity of the Peritoneum.* By M. Hourmann.

A patient who was in the hospital of Loursine under M. Hourmann, labouring under leucorrhœa, was ordered an injection into the uterine cavity. It was administered by means of a clyster pump, and at the first stroke of the piston the patient uttered a sharp cry, and pressed the hand strongly on the left iliac region. Symptoms of metroperitonitis came on and the most energetic treatment was adopted. It being an important practical point to determine whether peritonitis was likely to ensue from uterine injections, several novel experiments were made in the hospital of Loursine to determine whether liquids would pass along the Fallopian tubes into the cavity of the peritoneum; and it was found that injections with an arterial syringe passed into the tubes. Abundant injections by means of syringes double the size of urethral syringes passed into the tubes preceded by a certain quantity of air. In some few cases small quantities passed quite through the tubes. As this matter is to undergo further examination, we shall defer further notice of it at present.

*Gazette Médicale. Juillet* 11, 1840.

---

### *Congenital Dislocation of the Humerus reduced after Sixteen Years.* By M. Gaillard, Surgeon to the Hôtel Dieu, of Poictiers.

The following is an abstract of a report to the Royal Academy of Medicine, drawn up by MM. Cannet and Bouvier. They consider the case as very curious, and without a parallel in the history of surgery.

Mademoiselle L. B. a few days after birth presented a deformity of the left superior extremity with much pain during its movements. The elbow was thrown from the body, the forearm semiflexed, and the hand in a state of pronation. During her infancy she could not bring the elbow near the trunk, nor raise her hand higher than the chin. The limb was often painful during her early years; but latterly the pain had only come on when the limb had been long fixed in one position. When M. Gaillard saw her in 1836 she was sixteen years of age. He found in the posterior part of the shoulder a projection formed by the head of the humerus, which was placed in the subspinous fossa of the scapula, above the middle part of the spine, which was curved by the pressure it had undergone. On moving the arm, the head of the bone was felt rolling under the fingers, and it appeared abraded from rubbing on an irregular surface. The hand could not be brought to a state of supination. The forearm could not be extended on account of the permanent contraction of the biceps. Elevation and rotation of the arm were impossible.

On the 5th of January, 1837, the patient was placed on a stool, a cushion was applied to the external border of the scapula, and maintained by two cords passing from its extremities and attached to two fixed rings. The arm directed horizontally inwards was then submitted to the action of a weight of sixteen pounds. The weight was suspended at the end of a cord which passed through a fixed pulley, and was attached to a bracelet fixed above the elbow. Time after time M. Gaillard increased the power of the traction, occasionally adding his efforts to the force exercised by the weights. The extension prolonged from twenty to twenty-five minutes was repeated on the 10th and 11th of January, each time bringing the head of the humerus nearer the glenoid cavity, and with no other inconvenience than a slight pain and swelling of the arm. On the 13th the head of the humerus was more moveable. Further traction was employed, the arm being always placed horizontally. The humerus yielded, glided over the scapula for the space of an inch and a half, and approached the glenoid cavity. M. Gaillard then seized the elbow, and by carrying it backwards and upwards he directed the head of the humerus downwards and forwards, then lowering the limb he felt the head pass under the acromial arch, and leap over a projection which appeared to belong to the articular cavity. The arm was now in contact with the trunk, it was sensibly lengthened, and all its movements could be executed with ease.

The dislocation, however, recurred again and again, and was as often reduced. The difficulty was to maintain the bone after reduction. This, however, was effected by careful bandaging. The patient suffered considerable pain at times, but was relieved by the application of leeches. From the first reduction to the entire cessation of pain a period of more than two years elapsed; but the cure was then complete, the patient using the limb equally as well as the other.

*Bulletin de l'Académie Royale de Médecine. Juin* 30, 1840.

---

*The Myopodiorthoticon, or Apparatus for the Cure of Shortsightedness.* By Professor A. A. BERTHOLD, of Göttingen.

THE principle of this apparatus (whose long name is derived from *μυωψ*, one that is shortsighted, and *διορθωτικον*, having power to correct,) is founded on the possibility of increasing by exercise the power of accommodating the eyes for clear vision at different distances. Myopia depends, it is well known, on the rays of light which enter the eye being brought to a focus too soon, and in front of the retina, on which exactly the image should be formed. On whatever physical peculiarity in the eye this defect may depend, it is believed that it might be compensated by an increase of that voluntary power of altering the form of the eye, which all exercise in looking alternately at near and at distant objects.

The whole of the apparatus, except a few screws, is made of wood. It consists of a reading desk which may be set on any table, and which is about a foot and a half long and a foot wide. This desk is moveable by hinges on its anterior side, or by some other means on a board of about the same size, so that it may be placed in a convenient position, and be fixed there by a stop attached to the board. From the back part of the desk a screw goes straight upwards on each side; and these screws pass through a moveable cross-piece, which can be either raised up or drawn down and fixed. Through the middle of this cross-piece, there is a horizontal hole, for the passage of a bridge which is directed forwards and a little upwards, and which in front is hollowed out to receive the upper part of the root of the nose. On its upper surface this bridge is graduated in inches and lines, and it is moveable in the hole through the cross-piece, and can be fixed in it by screws. By the mobility of the desk on the board, of the cross-piece on the upright side-screws, and of the bridge in the cross-piece, the apparatus may be arranged so that the book which is to be read may have the best possible position in relation to the eye. By the side a measuring rod passes from the desk upwards through a hole in the cross-piece, so as to determine with the assistance of the measure on the bridge the gradual decrease of the myopia, and to enable the apparatus to be fixed in regularly determined positions, according to the size of the print which is being read.

The author relates a case of most aggravated myopia, in which the patient, a man twenty-six years old, who had never been able to read at a greater distance than five inches, was enabled, by the use of this apparatus for about four months, to read just as easily at a distance of eleven inches and four lines. The distance between the book and the rest for the nose was increased from half a line to a line every two or three days by means of the screws; and the author believes from what he saw in this case that slowness is essential to certainty of cure.

*Von Ammon's Monatsschrift. June,* 1840.

---

*Luxation of the Second Cervical Vertebra, reduced after seven months by peculiar method.* By M. JULES GUERIN.

THE author, in a letter to the president of the Royal Academy of Sciences, states that this case resulted from a fall on the chin; but the displacement did not occur till the next day, and he attributed it then to muscular contraction. On this view of the mechanism effecting the luxation, he has employed an analogous mode of reduction, but in the opposite direction; the displacement of the vertebra being consecutive to the rupture of the ligaments, and of part of the articular surface, under the influence of certain muscles, he endeavoured to bring the antagonist muscles into play and thus gradually produce replacement. This was effected without accident, and the appearances of the luxation successively disappeared; and the young woman after three months consecutive treatment has the neck perfectly straight, and can execute any movement of the head with the greatest facility. This is interesting, as there is much difference of opinion among surgeons as to the propriety of attempting reduction in such cases. In this instance MM. Marjolin and Bouvier thought there would be great danger in the attempt.

*L'Expérience.* *Juin* 18, 1840.

---

*New Operation for Prolapsus Ani.* By M. Robert.

Relaxation of the sphincter ani being the cause of this disease, all the remedies hitherto employed for its cure are inefficacious when it arrives at the last stage, as they can only act on the mucous membrane of the rectum. These reflections have induced M. Robert to shorten the sphincter in proportion to the amount of relaxation, so that the two cut surfaces of the muscle might unite and form a narrow ring to oppose to the descent of the mucous membrane. This operation was performed with success on a washerwoman, thirty-three years of age, in June 1839, in the hospital of La Pitié. This woman in her third pregnancy had a prolapsus ani, which was only temporary though it caused some pain. Her fourth pregnancy produced a prolapsus uteri, a permanent and considerable prolapsus ani, with relaxation of the abdominal walls. M. Robert excised a portion of the mucous membrane with some temporary relief; but the disease afterwards increased, the discharge of fæces became involuntary, and she suffered from pains in the loins and upper part of the thighs. When she entered the hospital the sphincter was so much relaxed that four fingers could be easily introduced.

The patient having been prepared for the operation by progressive diminution in diet, and the use of opium in order to effect long-continued constipation, M. Robert proceeded to operate in the following manner: An incision was made on each side of the anus, each incision being commenced a few lines external to the orifice, and carried backwards towards the coccyx. The fold of integument between the incisions, together with the portion of sphincter it covered, were removed, and the muscle was thus shortened by half its length. The wound was united from one side to the other by three points of suture. On the sixth day after the operation the sutures were removed. Union was nearly complete, but a fistulous passage remained from the anus to the coccyx. On the fifteenth day the woman had not passed any fæces. On the next day, the want of defecation being felt, in order to prevent any straining, the bowels were relieved by the curette. On the forty-first day the patient, who before the operation could not retain her fæces, kept an injection during the whole day; there was no more prolapsus; the opening had become of the ordinary size; but the finger when introduced did not experience the energetic contraction of the sphincter which occurs in the normal state. With the exception of a slight protrusion of mucous membrane the cure was complete in August.

*Gazette Médicale.* *Juin* 20, 1840.

---

*Two Cases of the Excision of the entire Clavicle: one operation performed in* 1835 *by Dr. Cajetan Mazzoni, of Pisa, the other by Professor Charles Biagini, of Pistoia.* Communicated by Dr. Aronsohn, of Strasburg.

When we consider the situation of the clavicle and the important offices it fulfils, it is natural to suppose that its loss must be followed by severe inconveniences, and that the form of the chest and shoulder with the motions of the arm would be necessarily injured. Experience, however, in this as in other cases shows the futility of *à priori* reasoning in medicine.

CASE I. The first case was one of necrosis of the clavicle in a boy four years of age, of a decidedly scrofulous habit. About the time when spontaneous separation of the necrosed portions of bone was commencing, Dr. Mazzoni considered that the time had arrived to assist nature by extraction. Believing that the attachments of the clavicular muscles were destroyed, not only because necrosis was made evident by the probe, which proved that the periosteum was gone, but also because the bone was moveable; Dr. Cajetan thought it only necessary to divide the ligaments situated at the articular extremities of the bone. A director was introduced into a fistulous opening which existed near the sterno-clavicular articulation and a bistoury being passed along it the integuments were divided: the clavicle was seized with a pair of forceps, and the costo-clavicular ligament was divided with a straight blunt-pointed bistoury. Afterwards the other ligaments uniting the clavicle to the sternum were divided. The acromial extremity of the clavicle was apparent through an ulcer of the integuments, and therefore this bone was easily detached from the scapula. The entire extraction was completed with little loss of blood, and a portion of integument between the two wounds remained entire. The cure was completed within a month.

Three years after the operation the patient was in perfect health. The shoulder deprived of the clavicle was slightly depressed and approaching the sternum, but without affecting the symmetry of the parts. The motions of the arm were unlimited in any direction, the patient readily climbing trees.

CASE II. A boy fifteen years of age was admitted into the hospital of Pistoia in August 1838 under M. Biagini. The clavicle was affected with scrofulous disease, and was removed in a similar manner to the former case. Slight hemorrhage followed; but was stopped with charpie and moderate pressure. Caustic was occasionally applied to the wound and it had cicatrized within thirty days. This case is peculiarly interesting, as a firm fibro-cartilaginous substance now takes the place of the lost clavicle, so that the loss is not felt, every motion being perfect.

[For other cases of a similar operation, one by Mr. Travers, the reader is referred to Vol. V., p. 147, and to page 265 of the present Volume of our Journal. These cases will be useful as auxiliary proofs of the success of surgical resource comparatively new, and thereby encouraging surgeons to operate rather than leave a patient to almost certain death.]

*Gazette Médicale. Juillet* 18, 1840.

---

*On the Radical Cure of Inguinal Hernia by the Horizontal Position, after the plan of M. Ravin.* By M. BIAGINI.

A man, thirty-two years of age, consulted M. Biagini on account of a tumour in the left inguinal region, which this physician considered to be an entero-bubonocele, and which had resisted all efforts at reduction. M. Biagini proposed a long continuance in the horizontal posture to the patient, aided by compression, but he refused it. A year afterwards the man was obliged to put himself under treatment for articular rheumatism complicated by pericarditis. After three months decubitus, which was consequent on the rheumatic affection and its treatment, the hernia did not exist, nor could it be made to appear by any effort or coughing. M. Biagini felt assured that the left external inguinal ring was singularly contracted. He considered this case as confirmative of the views of M. Ravin, and drew the following conclusions: 1. It is possible to obtain a radical cure of some herniæ by a long continuance of the horizontal position. 2. The cure takes place by a diminution in the size of the canal and the retraction of its walls, by which the lost obliquity is restored. These changes occur solely by the *vis tonica* of the tissues. 3. Though the tissues possess sufficient *vis tonica* to bring about the desirable changes, it is necessary that the general strength of system of the patient be somewhat considerable. *Bulletino delle Scienze Mediche. Gennario,* 1840.

*On the Operation for Strabismus.* By Professor DIEFFENBACH.

SINCE my first communication on this operation it has had such a general reception and has acquired such an importance as I did not at that time anticipate. Upwards of 300 cases have been operated on by me within a few months, and both in Berlin and in other places my proceeding has been frequently imitated. I propose to give here a short general view of the results of my observations.

The youngest individuals in whom I have undertaken the division of the shortened muscle of the eye were five years old; the oldest were upwards of forty.

Sometimes one, sometimes both eyes squinted, and the operation had generally the same favorable result in both cases. When both eyes were affected I either operated first on that which squinted most, and when that was quite well on the other, or else on both at the same time.

Squinting inwards, from shortening of the rectus internus, was by far most frequent. Sometimes the trochlearis muscle was also shortened, so that it was necessary to divide it as well as the rectus. In the whole number of those I operated on there were only a few who squinted outwards, and still fewer in whom the eye was directed upwards or upwards and inwards. I found no eyes at all that squinted downwards.

Strabismus upwards was sometimes complicated with blepharoptosis. The division of the rectus superior not only cured the squinting, but the ptosis gradually diminished after it.

Strabismus outwards or inwards was often complicated with nystagmus bulbi. After the division of the external or internal rectus not only did the squinting cease but in general the nystagmus also. In other cases, however, the latter was persistent, and did not decrease till after the division of the rectus superior, or obliquus superior, or rectus externus.

When cataract and strabismus coexisted, the operations for both were done at the same time, and the result was in every case favorable to both.

In most of the patients the strabismus had commenced in very early childhood after ophthalmia neonatorum, scrofulous inflammation of the eyes with ulcers on the cornea, or after acute exanthemata, &c. In many there were cicatrices on the cornea or cataracta centralis. In cases of the former kind, in which hitherto artificial pupils would have been made, the operation was attended by success and considerable improvement of the sight.

All those who had strabismus of only one eye saw more weakly with it than with the other; in those who squinted with both eyes that which was turned least was usually the stronger. The weakness of the one eye had been observed by only a few of the patients; they had naturally looked only with the better eye and the other had been unemployed. The operation completely cured the weakness of sight; some who had actually amaurotic amblyopia could see clearly directly after it was performed.

Some of the patients previous to the operation often saw double; this defect continued for some time after it and then gradually ceased. Some others who had never seen double before did so immediately after the operation. These had been in the habit of looking only with their strong eye while the other had been unused. The improved position of the latter compelled it to see; but the double vision was subsequently lost.

Some who were operated on did not see so well immediately after as before the operation; but after some exercise this weakness of vision ceased and they could then see quite clearly. The cause of this was that when the eye was put in its normal position a point of the retina which was before unexercised was now brought into play and required some practice before it could fully discharge its functions.

*Operation.* That for strabismus convergens is here taken as the type. The operator always stands on the right side of the patient whether he be operating on the right or on the left eye. The patient sits on a stool, and an assistant standing behind him draws up the upper eyelid with a Pellier's hook. A second assistant draws down the lower eyelid with a double hook which is set in a handle and of which the teeth are connected by a transverse piece. He kneels down before the patient so as not to be in the way.

The operator then puts a fine hook into the conjunctiva at the inner angle of the eye just where it is passing from the palpebræ to the bulb, passes it superficially through it, and gives it to a third assistant who stands on the left side of the patient. The operator next passes a second hook in the same way through the conjunctiva about a line and a half from the first. He and his assistant then both at the same time draw their hooks a little up, so as to raise a fold of the conjunctiva, and at the same time pull the bulb somewhat outwards. The fold is then divided with a pair of curved eye-scissors; and this cut usually at once exposes the tendon and the anterior part of the muscle. A couple of cuts with the scissors then expose the outer surface of the muscle; a rather blunt hook is passed under its tendon, and the two sharp hooks that held the conjunctiva are now removed; the eye is held completely in the power of the blunt hook, and is to be drawn by it from out the internal angle of the orbit. A flat probe is then pushed under the muscle; and the loose connexion by cellular tissue between it and the eye is broken up. The division of the muscle is made by the scissors already mentioned, either, first, through the tendon in front of the hook; or, second, behind the hook at the beginning of the muscular substance; or, third, some lines deeper back.

When the tendon is divided nothing of it remains on the eye, and the muscle commonly retracts a line backwards. When the muscle itself is divided at its anterior part or further back, its posterior portion retracts, and the anterior, which remains connected with the bulb, turns forwards like a loose flap, which according to circumstances, may be removed by the scissors or pushed back into the wound if it is thought desirable that it should unite again with the posterior portion.

In practised hands the whole operation seldom lasts more than a minute; and it is done almost without pain. When finished the eye is cleaned with cold water and a soft sponge. The after-treatment consists of cold lotions, and very great abstinence from food and strong drinks. The patient should be kept in a darkened room. In most cases the wound heals very quickly, and after a few weeks no traces of the operation remain, and the eye stands in its normal position.

The operation for internal strabismus is by far the most easy; the division of the obliquus superior for squinting upwards and inwards is more difficult; that of the rectus externus for strabismus divergens is more difficult still; and the most difficult of all is the division of the rectus superior for squinting upwards. With respect to the manipulations of these operations they are just the same as those for strabismus convergens.

*Remarks on the operation.* The fixing of the upper and lower eyelids with the elevator and the hook so as to expose the whole of the anterior surface of the globe is indispensable, for neither the will of the patient nor the separation of the lids by the finger can do this effectually.

The fixing of the globe can be accomplished only by fine hooks carried superficially through the conjunctiva; the seizing and elevation of the fold of conjunctiva by forceps sounds more gentle than to do it with a sharp hook, but it is in reality far more painful, more injurious, and more insecure; the fold raised up by the forceps easily tears or slips from their grasp, and if the forceps are made with hooks they wound as well as pinch the membrane. Two hooks must be employed to make the fold tense enough.

The great number of operations that I have performed has given me opportunity of observing the phenomena that ensue subsequently to them, and their after consequences. The question here is only of internal strabismus, but any surgeon will easily supply the necessary modifications for the operations in the other varieties. In the first case the eye after the division of the muscle goes into its normal position. In the second it remains in some degree squinting. In the third it turns outwards.

In my first operations the position of the eye after the division of the muscle was left to chance, but I gradually succeeded in getting it into my own power to determine it. If there be the slightest degree of convergent strabismus, only a very small opening should be made in the conjunctiva, and the tendon only divided close to the eye without separating the muscle from the globe. In this case the eye at first almost maintains its previous position, but after some weeks it becomes straight. If the conjunctiva be more extensively divided, and if the under surface of the muscle be separated from the globe with a probe and then cut across, the squinting is at once nearly or completely removed. If the conjunctiva be divided over a greater arc and towards the back of the globe, if the cellular tissue be extensively separated and the muscle be detached far back and divided at its middle, then the eye, even in cases in which the whole cornea was before hidden in the internal angle, stands quite straight after the operation.

Hundreds of those whom I have cured have been seen by our own and by foreign medical men, and it was often impossible to distinguish the eye that had squinted from the other.

In some who still squinted slightly after the operation the position of the eye was made perfectly normal by tying up the sound eye and rolling the globe forcibly outwards so as to stretch the newly-formed uniting substance. In others of those whom I first operated on, in whom I had not adopted this after measure, I repeated the operation; and I found that in fourteen days after its first division the muscle was again intimately united with the globe, and that with the exception of a slight thickening and induration of this part there was no indication of a previous operation.

Immediately after the operation the eye can be moved inwards by the superior oblique, and at a later period by the divided muscle which has again united with the globe. In several persons in whom the muscle was divided deeper in the orbit the eye turned outwards some weeks after the operation, so that there was actually an external strabismus. If the divergence was but slight it was often sufficient to snip (*betupfen*) the conjunctiva at the inner angle, so as by the shortening of the membrane that was thus produced, to bring the eye into the middle. But if the divergence was greater I then divided the external rectus and the eye became straight again, especially when I at the same time removed a fold of conjunctiva from the internal angle, for the cicatrix that then followed tended to draw the globe inwards. If the eye, notwithstanding the division of the external rectus, still remained turned outwards, I then, after dividing and separating that muscle, tied a thread as fine as a hair upon its tendon, and with this pulled the eye forcibly inwards. The end of the thread was then drawn tightly across the bridge of the nose and fastened to a piece of good adhesive plaster, which was stuck upon the opposite side. The result for the most part surpassed my expectations.

*Casper's Wochenschrift.* July 4, 1840.

---

### *On the Operation for Strabismus. Claims of M. Jules Guerin.*
### By M. Jules Guerin.

In a letter on the treatment of strabismus, addressed to the Academy of Sciences, 29th June, 1840, M. Guerin says: "I have the honour to inform the Academy that I have four times practised with success the division of the muscles of the eye, in cases of convergent strabismus. I shall briefly state the principles which have directed me in this operation. I had long and publicly professed that strabismus was the result of the contraction of the muscles of the eye; and the varieties of this deformity the necessary consequence of different degrees of contraction. It is in fact an application of my general theory of articular deformities in the skeleton, and caused one of the most eminent members of the academy to designate strabismus as *club-foot of the eye.*"

M. Guerin goes on to state that in his clinical lectures he frequently proposed to extend division of the muscles to deviations of the eye in the same manner that he had applied it to deformities of similar origin, and that eighteen months since he offered to cure Dr. Pinel Grandchamp of strabismus by means of this operation. He proceeds : "As for the operation itself it differs in some respects from that of M. Dieffenbach. One of the reasons which induced me to pause before attempting his method, was the fear of inflammation occurring in a wound exposed to the air upon a delicate organ, and so near the cerebrum. These accidents I feel certain of avoiding by the following method:

"In place of dividing layer after layer, I detach the portion of ocular conjunctiva, which covers the muscles from the sclerotic, and raise it with broad-nibbed forceps until the muscle is exposed. This being divided with curved scissors, I restore the detached portion of conjunctiva to its place; this by closing the wound prevents the access of air, and obtains all the advantages of a subcutaneous wound. Experience has confirmed my preconceived notions of the theory ; in the four operations I have performed there has been no sign of suppurative inflammation.

"The result of these operations has been very satisfactory ; but not so immediately advantageous as M. Dieffenbach observed. In one instance there was a complete and instantaneous restoration of the natural position of the eye, in the others only an amelioration. This circumstance appears to be a natural consequence of the true origin of strabismus. At one time the deviation of sight is originally muscular, and the result of spasmodic contraction in a single muscle; at another time the contraction is consecutive or even primitive; but it has simultaneously affected many muscles."

*L'Expérience. Juillet* 9, 1840.

---

*On the Cure of Strabismus.* By Dr. F. A. VON AMMON.

[IN the great number of the papers written on this subject, it is scarcely to be expected that more than two or three remarks on each should be worth abstracting. In the present article by the editor of the Monatsschrift, the following passages are worthy the attention of our readers :]

A principal point is the determination of the indications for the operation ; and I think it the more important, because the whole history of the affection, notwithstanding the works of many excellent physiologists, is extremely obscure and uncertain. Nor could this be otherwise when the anormal position of the eye is in different cases so varied, and so often the consequence of diseased conditions of the brain, the abdominal nervous system, and of the globe itself. Here is a wide range for the production of the disease independently of the cases which are produced by diseases of the orbit. No rational physician will expect to cure such a strabismus by myotomy; and the operation can be applicable only when the malposition of the eye is primarily dependent on the muscle, i. e., when there is a shortening of one of the muscles of the eye, which holds the eye to one side and limits its motions towards the other; but even here more than one condition must be laid down.

But unfortunately the necessary basis of the operation, pathological investigation, is at present absolutely and entirely deficient, no one has yet clearly convinced himself on the dead body that a shortened state of the muscle exists in squinting. The operation, however, may itself be made the means of investigating the nature of the disease; and here again, as has been the case with club-foot and wry-neck, surgery may be made the assistant of pathology.

With regard to the true orthopædics of the eye, i. e., its acquiring the right position after the operation, this consists in the constant endeavour and active exertion of the patient, to give his eye the proper direction. A club-foot or a wry-neck would just as soon return to its right position after division of the tendons without assistance as a squinting eye will. A good method of insuring success is to tie up the sound eye; the eye that has been operated on then always moves to the centre of the orbit, and the longer it is kept there the more likely it is to remain. In few cases will this or some similar measure be unnecessary.

The author also suggests the propriety of endeavouring to make a subcutaneous or rather a subconjunctival division of the muscle, which he believes would avoid all the inconveniences to which the present method is subject. He has performed it on the dead subject and on animals; and has no doubt it may be applied to actual cases of strabismus.

*Monatsschrift für Medicin, &c. June,* 1840.

---

*On certain Abscesses occurring in different parts of the Body, during the course of Diseases of the Urinary Organs.* By M. Civiale.

M. Civiale has observed that, sometimes during the progress of gonorrhœa and other diseases of the urethra, also in affections of the prostate gland and when there is stranguary or dysuria, or foreign bodies in the bladder, certain parts of the body, particularly the extremities and the larger joints, are attacked with pain, which may be dull and diffused, or acute and circumscribed, which is at first generally supposed to be rheumatic, but which terminates in the formation of large abscesses, differing in some respect from common phlegmons. M. Civiale states that these cases have escaped the observation of authors, but says that since his attention has been directed to them he has met with numerous examples. When these accidental pains first make their appearance, the patient complains of a peculiar "*empâtement*" (?) and numbness of the part, which come on before any swelling is apparent. These local symptoms are accompanied with lassitude, weakness, anxiety, and general constitutional disturbance which does not correspond at all with the degree of pain, which in the early stages is generally slight and diffused. As the case advances the pain becomes more acute, there is complete loss of appetite and sleep, great prostration of strength, the pulse being sometimes scarcely to be felt; rapid emaciation, dry tongue, troublesome cough, and lastly delirium. The disease often commences with a febrile paroxysm, which may return at regular intervals or be continued. Together with these symptoms the urine is often of a deep orange colour, and is excessively fetid, and when this state of the renal secretion is met with, the case is always very serious. Local redness and swelling of the painful part, with evidence of the formation of matter, frequently do not make their appearance till a late stage of the disease.

One peculiarity in this affection is a tendency in the local disease to shift its position, and it often appears in different places at the same time. The suppuration in distant organs is often accompanied also with the formation of an abscess in the scrotum, perineum, or neighbourhood of the urethra or bladder, which, however, form no communication with the urinary passages.

With regard to the causes of this disease, M. Civiale has not remarked any predisposing circumstances. All the cases occurred in adults. In four there was stricture of the urethra, during the treatment of which affection, (in one case, by cauterization, and in the three others by dilatation,) the abscesses were developed. In a fifth case there was serious disease about the neck of the bladder, and in several others there was urinary calculus, which had been broken down by the operation of lithotrity. In all the cases there seemed to have been some considerable irritation or inflammation of the urethra, or neck of the bladder, which appeared to give rise to the distant affection.

The peculiar state of the system, accompanied by the formation of these abscesses, is attended with considerable danger, and M. Civiale lost the first two patients which he treated.

The treatment found most efficacious was scarification of the whole of the affected surface in the early stage (from which a reddish or yellow coloured serum is generally discharged for several days in considerable abundance,) and then the use of emollient poultices. This plan will sometimes cut short the disease, but if the inflammation has reached the deep-seated cellular tissue of the limb between the muscles, this texture becomes indurated, and a vast abscess soon forms; several large deep incisions must now be made completely through the affected parts which will allow of the escape of the matter, and also unload the distended vessels. The surgeon must not always wait for the sense of fluctuation to be apparent, before he makes his incisions, for great relief will be afforded by cutting through the inflamed and indurated tissues before pus has actually been secreted, and the progress of the disease may be thus arrested. M. Civiale does not make any remarks concerning the general or constitutional treatment of the patient, but we presume that this must be conducted on common principles.

*Bulletin Gén. de Thérapeutique. Avril,* 1840.

---

*New Operation for the Cure of Vaginal Cystocele.* By M. JOBERT, Surgeon to the Hospital of St. Louis.

THE following is the substance of a report made to the Royal Academy of Medicine, by a committee consisting of MM. Blandin, Danyan, and Gimelle.

M. Jobert divides his memoir into three parts: in the first, he treats of the history of the disease, and the modes of treatment which have been hitherto employed for its removal. In the second, he proceeds to point out his method of obtaining a radical cure. In the third, he gives some new ideas, based on his pathological researches, with regard to the mode of formation of the cystocele.

Vaginal cystocele was treated of in the work of Leblanc, printed in 1775. Since that time, many surgical authors have noticed it; all have attributed it to a rupture or abrasion of the anterior wall of the vagina, allowing the bladder to pass into the cavity of the former, filling it by its distension, and causing a projection more or less considerable through the os externum. Until lately, palliative means alone have been adopted, which were totally incapable of effecting a radical cure. Pessaries under all forms can be but palliatives.

The well-known operations of Marshall Hall and Dieffenbach have been performed with success by Berard and Velpeau in cases of prolapsus uteri, in which the bladder and vagina have been also drawn down; by these means the displaced parts may be retained within the cavity of the vagina; but in cystocele, M. Jobert says the bladder has an abnormal magnitude, and although it does not project from the vulva, it is nevertheless displaced, resting in such a manner on the perineal surface of the vagina, that the anterior wall of the bladder becomes superior. Thus after the operation just named, though there is not external projection, the organ is not able to regain its ordinary size or position.

M. Jobert considers that in order to effect a radical cure, three things must be done: first, the bladder must be restored to its natural situation; second, the anterior wall of the vagina distended by the tumour must be reduced to its ordinary proportions, so as to maintain the bladder when replaced; but this latter result cannot be obtained by causing a loss of substance in this wall, and reuniting the edges according to the method of Hall, without injuring the orifice of the vulva. The proceeding has also entailed great difficulties in its execution, and possibly severe accidents, therefore the author substitutes, thirdly, the following method: He incloses within two curved transverse lines an oval space, more or less considerable, on the posterior surface of the tumour or the anterior surface of the vagina by means of caustic, so as to form an isolated spot, repeating the application of the caustic until the mucous membrane is destroyed. He then pares the edges with scissors or a bistoury, draws them together, and maintains them in apposition by means of straight needles, the points of which are removed, and a twisted suture.

Jobert operated in this manner on the 23d of July, 1838, on a female aged forty-five, of strong constitution, who presented at the aperture of the vulva a very large cystocele, which elevated the nymphæ and descended above the urethra and clitoris. The tumour was reddish. The patient easily returned and reproduced it. She often experienced acute pains in the abdomen, and the tumour became excoriated from friction. She had frequent desire to pass urine, the emission of which was often difficult. The tumour was five inches in length, from the orifice of the urethra to the neck of the uterus, and eight inches in circumference.

Seven needles were applied; four large and three small ones. They were lance-shaped, and were contained in sheaths, which alone were left to retain the threads when the suture was made. One of the large needles was applied to each extremity of the oval, formed by the circumscribed space, two others in the middle, the small ones filling up the intervals. A sound of gumelastic was introduced into the bladder and left there in order to prevent the accumulation of urine, and the distension of the viscus. The reunion of the edges of the wound arrested the flow of blood, and no accident occurred. Four of the sheaths of the needles which retained the sutures were removed on the 3d of August, the others fell of themselves on the 5th and 7th. The sound was discontinued on the 20th of the same month. The woman returned to her occupation as a laundress, and at the end of the month of January, 1839, the time at which she was examined, nothing unfortunate had happened.

Since this time, two other cases presenting the same symptoms as that just related were operated upon in the same manner by M. Jobert, and the result in all the cases has been equally favorable. Two of the committee appointed by the academy were able to testify to this in one case.

But after the cure of these patients, the anterior wall of the vagina underwent a contraction throughout its whole length, and the uterus was proportionally prolapsed. M. Jobert now prevents this inconvenience by applying the caustic in a vertical instead of transverse direction. In this way the vagina is retained, and remains of its full length, the uterus suffering no displacement.

Jobert has shown by his pathological researches that the anterior wall of the vagina is neither ruptured nor abraded in the displacement of the bladder called cystocele, but that this disease is the result of the relaxation of the superior pelvic fascia, which having been distended by the elevation of the womb in successive pregnancies, loses its elasticity and no longer sustains the anterior wall of the vagina after delivery in a proper manner; and in this case, a fall, an effort, the habit of retaining the urine in the bladder for too long a period and in too great a quantity, are causes sufficient to produce the disease.

Jobert performed the same operation with success in a case of eversion of the mucous membrane of the posterior wall of the vagina; and the committee are of opinion that it may be practised in similiar states of the same tissue in other natural passages.

*Bulletin de l'Académie Royale de Médecine.* Mai 15, 1840.

---

### *On the Etiology and Treatment of Congenital Luxations and Pseudo-Luxations of the Femur.* By Dr. J. Guerin.

I have established it as a fact, in my History of Deformities of the Osseous System, that the greater number of congenital articular contractions are the product of primitive muscular retraction. . . . . . . . . . . . . I conclude: 1. That congenital luxations of the hip-joint are, like club-foot, torticolis, and spinal deviations, the product of primitive muscular retraction, and that the kind of luxation depends upon the direction in which the retraction happens. 2. There exists an order of deformities of the hip-joint not hitherto indicated, and to which I apply the term *pseudo-luxations,* because they look like luxations, although the head of the bone remains in the cotyloid cavity. These vary as do the complete luxations, and from the same causes. 3. The essential treatment consists in division of the retracted muscles. I have thrice performed this operation with success. The cogenital luxations and pseudo-luxations of joints depend in the majority of cases on primitive muscular contraction, which takes place in three different modes: by shortening, by paralysis, and by stoppage of development of the retracted muscles; and the different varities of these deformities are, like those of the neck, the spine, and the foot, the effect of this retraction differently distributed in the muscles of these parts. In a girl aged fourteen years, I divided the biceps, semi-tendinous, semi-membranous, and rectus internus, for two incomplete luxations of the knee, produced by primitive retraction of these muscles; on each side there was subluxation of the tibia backwards upon the condyles of the femur, rotation of the limb outwards through the space of about one fourth of a circle, and an inclination outwards of the leg upon the thigh of about sixty degrees. The rotation outwards, the lateral inclination and the gliding backwards of the tibiæ, were reduced on the day following the operation to the simple normal flexion of the leg upon the thigh, and since that period there remained nothing more than a certain degree of permanent flexion of the joint. In order to convince those who feel any doubt respecting the safety with which these operations may be performed, I may add that, on the same day, I successively divided in the young female above mentioned thirteen muscles or tendons beneath the skin, for the relief of certain deformities from which she suffered; on the following day she felt no kind of pain nor indisposition, nor was there any symptoms of inflammation in any one of the divided parts.

*Gazette Médicale de Paris.* No. iv. 1840.

---

*Therapeutic Considerations on Obliterations of the Veins in the Treatment of Varix and Varicose Ulcers.* By M. JOBERT.

IN this paper M. Jobert relates six cases, and states that he could describe twelve others, in all of which the treatment of varicose veins by producing the obliteration of their trunks by needles and twisted sutures, or other means, though at first it seemed promising, had ultimately proved completely unsuccessful. In all the patients who were thus treated the varicose ulcers returned a short time after they left the hospital, as it was believed perfectly cured; and the return took place in these cases more rapidly than in all probability it would if only the ordinary treatment by rest, poultices, and bandages had been pursued.

[After the statement of these facts and those recorded byBonnet,(Brit. and For. Med. Rev., No. IX, p. 252,) few will hesitate to agree with the author that the obliteration of the veins by the needle and suture, however harmless an operation it may be, is not one which the surgeon has any authority or encouragement in undertaking in the treatment of varicose ulcers. The histories of the cases hitherto published in this country have not extended to the period after the operation at which the fair promises that it affords at first are dispelled.]

*Bulletin de Thérapeutique. Mai,* 1840.

